# Critical and diverse role of alarmin cytokines in parasitic infections

**DOI:** 10.3389/fcimb.2024.1418500

**Published:** 2024-11-04

**Authors:** Zhou Xing, Suiyi Liu, Xing He

**Affiliations:** ^1^ Department of Tropical Diseases, Naval Medical University, Shanghai, China; ^2^ Department of Medical Engineering, Shanghai Eastern Hepatobiliary Surgery Hospital, Naval Medical University, Shanghai, China

**Keywords:** IL-25, IL-33, TSLP, type 2 immunity, parasitic infection

## Abstract

Alarmin cytokines including IL-25, IL-33, and thymic stromal lymphopoietin (TSLP) function as danger signals to trigger host immunity in response to tissue injury caused by pathogenic factors such as parasitic infections. Parasitic diseases also provide an excellent context to study their functions and mechanisms. Numerous studies have indicated that alarmin cytokine released by non-immune cells such as epithelial and stromal cells induce the hosts to initiate a type 2 immunity that drives parasite expulsion but also host pathology such as tissue injury and fibrosis. By contrast, alarmin cytokines especially IL-33 derived from immune cells such as dendritic cells may elicit an immuno-suppressive milieu that promotes host tolerance to parasites. Additionally, the role of alarmin cytokines in parasite infections is reported to depend on species of parasites, cellular source of alarmin cytokines, and immune microenvironment, all of which is relevant to the parasitic sites or organs. This narrative review aims to provide information on the crucial and diverse role of alarmin cytokines in parasitic infections involved in different organs including intestine, lung, liver and brain.

## Introduction

Parasitic infections are a major global health and social burden, impacting over 25% of the global population with many more at risk of infection (Ryan et al., 2020). The interplay between parasites and the host immune responses determines the outcome of such diseases. Parasite infections can elicit a type 1 immune response characterized by the elevation of T helper 1 (Th1) cytokines such as interferon-γ, or a type 2 immune response featured by the production of Th2 cytokines such as interleukin 4 (IL-4), IL-5, and IL-13 (Engwerda et al., 2014; Zaiss et al., 2024). Notably, some parasites simultaneously induce both type 1 and type 2 responses (Muñoz-Carrillo et al., 2017; Muñoz-Carrillo et al., 2021). Host immune response is crucial to drive parasite expulsion or killing, whereas strong and long-lasting immune response can contribute to the development of host pathology such as tissue injury and fibrosis (Muñoz-Carrillo et al., 2021). However, the mechanism of the initiation and maintenance of host immunity induced by parasitic infections remains largely unknown.

Alarmins are endogenous molecules which function as danger signals such as parasitic infections and are rapidly released into the extracellular milieu in response to tissue injury to trigger defensive immune responses (Maizels, 2020). Among them, IL-25, IL-33, and thymic stromal lymphopoietin (TSLP) are the first cytokines which are shown to activate group 2 innate lymphoid cell (ILC2) and have been conceptually grouped together ever since (El-Naccache et al., 2021). IL-25 (also known as IL-17E) belongs to the IL-17 cytokine family, which is produced by epithelial and immune cells. Its receptor IL-25R is a heterodimer consist of the common IL-17RA chain and specific IL-17RB chain (Wu et al., 2022). IL-33 is a member of the IL-1 family of cytokines, which is primarily expressed by non-immune cells such as epithelial cells, endothelial cells, and fibroblasts. Unlike the other cytokines, IL-33 is localized in the cell nucleus through its N-terminal domain which contains a chromatin-binding motif. The nuclear IL-33 can be passively released as alarmins from necrotic cells during tissue damage and unconventionally secreted from living cells. After released from cells, IL-33 binds to its receptor, suppressor of tumorigenesis 2 (ST2), on target cells (Dwyer et al., 2022). TSLP belongs to the IL-2 family of cytokines, which is mainly expressed by the epithelial cells of the gut, lung, and skin. Its receptor is a heterodimer of the common IL-7RA, which is shared with IL-7, and the specific TSLP receptor (TSLPR) (Corren and Ziegler, 2019). The importance of these alarmin cytokines in immune response especially the type 2 immunity is widely documented and extends beyond their role in ILC2 biology, because they are key regulators of Th2 cells, macrophage, mast cells, basophils, dendritic cells (DCs), regulatory T cells (Tregs), and more (Stanbery et al., 2022).

Recent works have highlighted the release of alarmin cytokines from damaged or stimulated epithelial and stromal cells is crucial for the initiation and maintenance of host protection but also tissue pathology after parasite infections (Maizels et al., 2012; Hung et al., 2013). Parasitic diseases also provide an excellent context to study the functions and mechanisms of alarmin cytokines (Henry et al., 2017; He and Pan, 2018). By understanding how they instruct hosts to activate innate and adaptive immune cells in response to parasitic invasions, we may be able to uncover novel strategies to prevent or treat parasitic diseases and other diseases. In addition, growing evidence has indicated that the role of alarmin cytokines in parasite infections is dependent upon species of parasites, cellular source of alarmin cytokines, and immune microenvironment, all of which is relevant to the parasitic sites or organs (McSorley and Smyth, 2021). Therefore, this narrative review aims to provide information on the crucial and diverse role of alarmin cytokines in parasitic infections involved in different organs including intestine, lung, liver and brain. In the literature review process, we performed a systematic search on Google Scholar and PubMed with the keywords including “IL-25”, “IL-33”, “TSLP”, and “parasite”.

## Roles of alarmin cytokines in human diseases

It well documented that alarming cytokines are important regulators of type 2 inflammatory diseases triggered by parasitic worms and allergens (Liew et al., 2016; Gauvreau et al., 2023). In this review, we mainly summarized their crucial and diverse roles of in parasitic infections (**Table 1**), which will be addressed in detail in the subsequent sections. Since its link to atopic dermatitis, TSLP has widely been regarded as a promoter of type 2 inflammatory diseases, encompassing asthma, chronic rhinosinusitis, allergic rhinoconjunctivitis, and eosinophilic esophagitis (Rothenberg et al., 2010; Harada et al., 2011; Miyake et al., 2015; Ebina-Shibuya and Leonard, 2023). Multiple studies have highlighted the significance of IL-33 in airway allergic diseases. IL-33 levels are associated with the clinical severity of asthma, and genetic variants of IL-33 have been linked to susceptibility to allergic rhinitis and asthma risk (Sakashita et al., 2008; Kurowska-Stolarska et al., 2009). Additionally, IL-25 has been implicated in various models of allergic inflammation, such as asthma and chronic rhinosinusitis (Divekar and Kita, 2015; Patel et al., 2019). On the other hand, emerging data indicate that alarmin cytokines are not only involved in canonical type 2 responses but are also important in the context of various human diseases and even homeostasis. The involved human diseases include viral and bacterial infections, cancers, metabolic diseases, fibrotic diseases, and *etc.* They influence these diseases through both type 2– and non–type 2–mediated mechanisms (Monteleone et al., 2010; Rostan et al., 2015; Nie et al., 2016; Kotsiou et al., 2018; Tu and Yang, 2019).

**Table 1:** Summary of the role of alarmin cytokines in parasitic infections.

Parasitic site	Functional response	Alarmin	Parasite species	References
Intestine	Initiating parasite expulsion	IL-33	*N. brasiliensis, H. polygyrus, Trichuris muris, S. ratti, E. histolytica*	(Coakley et al., 2017; Meiners et al., 2020; Chen et al., 2021; Stanbery et al., 2022; Uddin et al., 2022)
		IL-25	*N. brasiliensis, H. polygyrus, Trichuris muris, T. spiralis, E. caproni, E. histolytica*	(Owyang et al., 2006; Zaiss et al., 2013; Muñoz-Antoli et al., 2016; Angkasekwinai et al., 2017; Noor et al., 2017; Varyani et al., 2022)
		TSLP	*Trichuris muris, T. spiralis*	(Massacand et al., 2009; Giacomin et al., 2012)
	Promoting host tolerance to parasites	IL-33	*N. brasiliensis*	(Hung et al., 2020)
		IL-25	*N. brasiliensis*	(Martin et al., 2018)
	Driving adaptive small intestine remodeling	IL-25	*H. polygyrus, Tritrichomonas muris*	(Schneider et al., 2018)
Lung	Initiating larvae killing	IL-33	*N. brasiliensis*, *S. venezuelensis*, *L. sigmodontis*	(Yasuda et al., 2012; Bouchery et al., 2015; Lenz et al., 2024)
	Accelerating pulmonary pathology	IL-33	*S. venezuelensis*, *L. sigmodontis*	(Araujo et al., 2016; Lenz et al., 2024)
		IL-25	*S. massoni*	(Hams et al., 2014)
Liver	Promoting the growth of parasite	IL-33	*E. multilocularis*	(Autier et al., 2023)
	Promoting the survival of parasite	IL-33	*S. japonicum*, *L. donovani*	(Rostan et al., 2013; Yu et al., 2015)
	Accelerating hepatic pathology	IL-33	*C. sinensis, S. massoni*	(McHedlidze et al., 2013; Yan et al., 2022)
	Inhibiting hepatic pathology	IL-33	*S. massoni, S. japonicum*	(Bai et al., 2021; Maggi et al., 2021)
Brain	Initiating parasite killing	IL-33	*T. gondii*	(Still et al., 2020)
	Accelerating cerebral pathology	IL-33	*P. berghei*	(Palomo et al., 2015; Reverchon et al., 2017)
	Inhibiting cerebral pathology	IL-33	*P. berghei, T. gondii*	(Jones et al., 2010; Besnard et al., 2015)

## Roles of alarmin cytokines in intestinal parasitic infections

Intestine is the most common colonized site for parasites. It is well documented that alarmin cytokines, especially for IL-33 and IL-25, are crucial protective regulators for hosts infected by intestinal helminths or protists. These intestinal helminths include *Nippostrongylus brasiliensis* (*N. brasiliensis*) (Stanbery et al., 2022; Varyani et al., 2022), *Heligmosomoides polygyrus* (*H. polygyrus*) (Zaiss et al., 2013; Coakley et al., 2017), *Trichuris muris* (Owyang et al., 2006; Chen et al., 2021), *Strongyloides ratti* (*S. ratti*) (Meiners et al., 2020), *Trichinella spiralis* (*T. spiralis*) (Angkasekwinai et al., 2017), and *Echinostoma caproni* (*E. caproni*) (Muñoz-Antoli et al., 2016). The intestinal protist includes *Entamoeba histolytica* (*E. histolytica*) (Noor et al., 2017; Uddin et al., 2022). Global IL-33 or IL-25 signaling deficiency resulted in delayed intestinal parasite expulsion, while administration of exogenous IL-33 or IL-25 led to accelerated intestinal parasite expulsion (Owyang et al., 2006; Zaiss et al., 2013; Muñoz-Antoli et al., 2016; Angkasekwinai et al., 2017; Coakley et al., 2017; Noor et al., 2017; Meiners et al., 2020; Chen et al., 2021; Stanbery et al., 2022; Uddin et al., 2022; Varyani et al., 2022). The extent of functional redundancy between IL-33 and IL-25 for intestinal parasitic infections is still unclear, but experiment evidence indicated that IL-33 and IL-25 may largely exert a co-operative action (Neill et al., 2010). The absence of both IL-33 and IL-25 signaling severely impaired *N. brasiliensis* expulsion, and their effector cells, ILC2, failed to expand in either the mesenteric lymph nodes (MLNs) or peritoneal lavage (Neill et al., 2010). However, TSLP is only essential for the expulsion of *Trichuris muris* and *T. spiralis* (Massacand et al., 2009; Giacomin et al., 2012).

**Figure 1:**
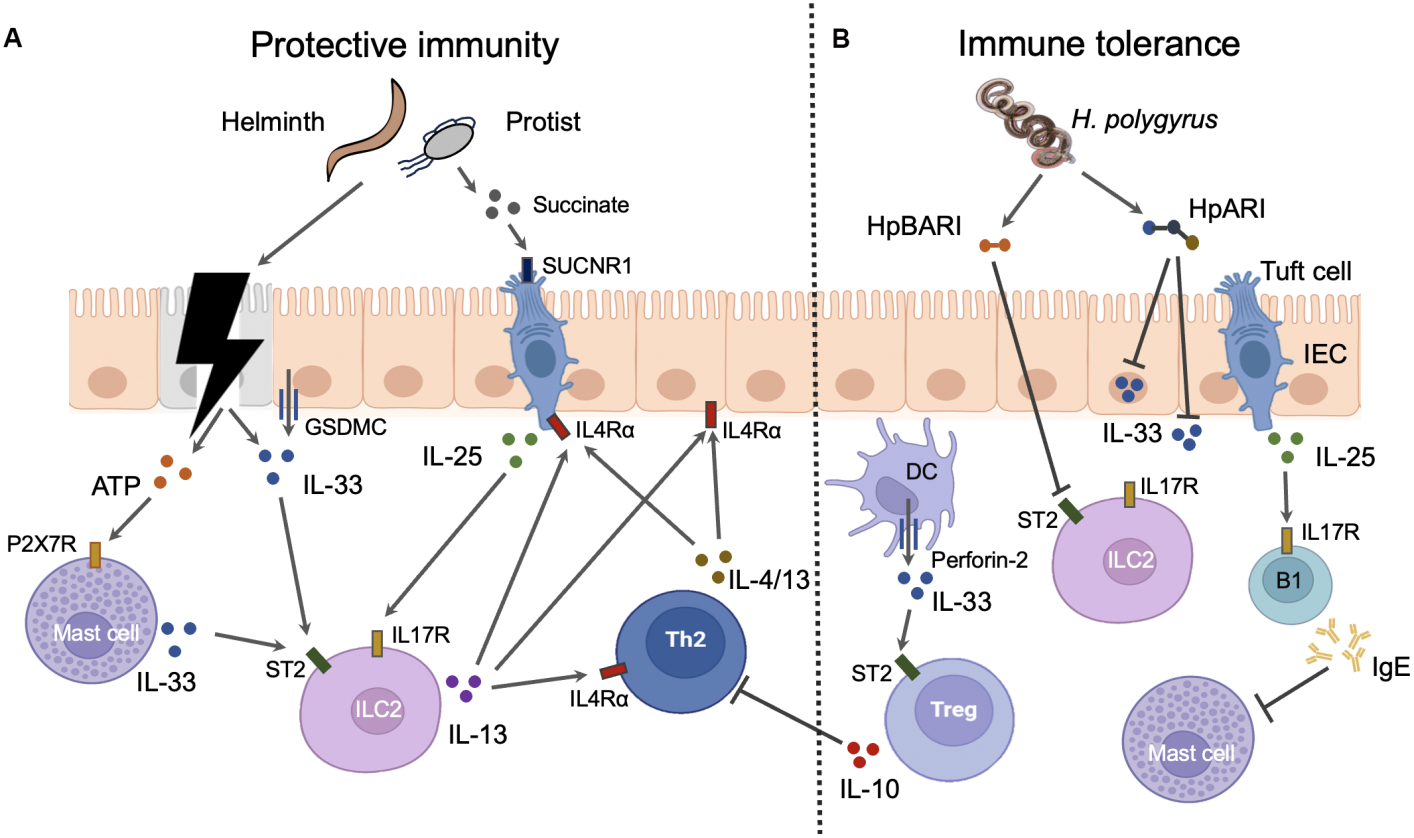
The roles and mechanisms of alarmin cytokines in intestinal parasitic infections. IL-25 and IL-33 can elicit both protective immunity and immune tolerance in intestine during infections. **(A)** Protective immunity. Parasites can cause the damage of intestinal epithelial cells (IECs) which results in the release of IL-33 from these cells. The injured IECs also release ATP which induce the mast cells to secret IL-33 through binding to P2X7R. Moreover, the intact IECs can actively secret IL-33 via GSDMC membrane pores after the activation of IL4Rα signaling. Epithelial tuft cells are the sole source of IL-25 in the gut. The metabolites such as succinate from intestinal parasites especially some protist can trigger the tuft cells to secret IL-25 through interaction with taste receptors such as SUCNR1. Both IL-25 and IL-33 can activate group 2 innate lymphoid cells (ILC2s) which express IL-25 receptor IL17R and IL-33 receptor ST2. IL-13 released from activated ILC2s promotes the activation of Th2 cells which produce Th2 cytokines such as IL-4 and IL-13. Th2 cytokines from both ILC2s and Th2 cells instruct the gut mucosa to mount a “weep and sweep” response to expel gut parasites through IL4Rα signaling. Th2 cytokines can also amplify the response of IECs and tuft cell to parasite invasion through IL4Rα signaling. Thus, the positive feed forward loop comprised of IECs, tuft cells, ILC2s, and Th2 cells is the main host defense mechanism against intestinal parasites. **(B)** Immune tolerance. Myeloid cells such as DCs release IL-33 through the inducible transmembrane pore-forming protein perforin-2. DC-derived IL-33 activates the ST2 positive intestinal Tregs which suppress the activation of Th2 cells. IL-25 induces B1 cell IgE production which blocks the activation of mast cells. *H. polygyrus* has evolved several mechanisms to negate the function of IL-33. Soluble excretory protein *H. polygyrus* alarmin release inhibitor (HpARI) can blocks the function of IL-33 by binding the intracellular and extracellular IL-33. Another soluble excretory protein *H. polygyrus* binds alarmin receptor and inhibits (HpBARI) can bind membrane ST2 which prevents IL-33-ST2 interactions.

The protective effects of IL-33 and IL-25 for intestinal parasitic infections are largely dependent on the activation of ILC2 which express IL-33 receptor ST2 and IL-25 receptor IL17RB (Mathä et al., 2022). It has been observed that, in the mouse model (C57BL/6) ILC2 is widely distributed at the tissue level, particularly in MLNs, spleen, and liver. These cells expand robustly after infection with intestinal parasites such as *N. brasiliensis* or in response to exogenous IL-33 or IL-25, and they are the major innate IL-13 resources under these conditions (Price et al., 2010). Activation of ILC2s is sufficient for *N. brasiliensis* clearance in the mouse model (C57BL/6), even in the absence of adaptive immunity (Stanbery et al., 2022). ILC2 is a functional heterogeneous cell population, which can be grouped into natural ILC2 (nILC2) and inflammatory ILC2 (iILC2). nILC2 locates at barrier tissues which are crucial for tissue homeostasis and primarily IL-33-responsive, while iILC2 is not resided in peripheral tissues in the steady state but can be elicited at many sites by intestinal parasite infection or IL-25/IL-33 treatment (Huang et al., 2015; Miller and Reinhardt, 2020). IL-13 is believed to be the main downstream effector of ILC2. The accumulation of IL-13 production from ILC2 instructs epithelial cells to produce resistin-like molecule beta (RELMβ) and recruits eosinophils, which together result in parasite destruction (Stanbery et al., 2022). In addition, ILC2 may also collaborate with Th2 and Th9 cells for hosts to better clear the intestinal parasites such as *T. spiralis* (Angkasekwinai et al., 2017). These cells together instruct the gut mucosa to mount a “weep and sweep” response to expel gut parasites through IL4Rα signaling (Bąska and Norbury, 2022) (**Figure 1**). TSLP promotes for the development of protective Th2 responses upon infection with *Trichuris muris* through inhibition of IL-12p40 production from dendritic cell (DC). Excretory-secretory (ES) products from *H. polygyrus* and *N. brasiliensis*, but not *Trichuris muris*, were capable of directly suppressing DC production of IL-12p40, thus bypassing the need for TSLP (Massacand et al., 2009).

Intestinal epithelial cells (IECs) are the dominant sources of IL-33 in the gut, while epithelial tuft cells are the sole source of IL-25 in the gut. As a nuclear cytokine, the IL-33 release from IECs is proposed to be resulted from cell necrosis induced by intestinal nematodes. The necrotic IECs also release ATPs which activate mast cell to produce IL-33 through P2X7 ATP receptor (Shimokawa et al., 2017). Mast cells are a potent source of IL-33 during infection with intestinal nematodes such as *H. polygyrus* (Shimokawa et al., 2017). However, IL-33 can also be actively released from IECs in the absence of cell death during infection (Zhao et al., 2022). This process requires the O-linked N-Acetylglucosamine transferase which mediates the O-GlcNAcylation of STAT6. This modification allows STAT6 to facilitate the unconventional IL-33 secretion from IECs via GSDMC membrane pores which also mediate the secretion of IL-1β and IL-18 from myeloid cells after inflammasome activation (Zhao et al., 2022). Besides, modified STAT6 also drives the differentiation of IL-25-producing tuft cells through promoting Pou2f3 transcription (Zhao et al., 2022). Thus, the type 2 cytokines can amplify the response of IECs and tuft cell to parasite invasion through IL4Rα/STAT6 signaling. The positive feed forward loop comprised of IECs, tuft cells, ILC2, and Th2 cells is the main host defense mechanism against intestinal parasites. Besides, the taste receptor signaling plays a crucial role in directing tuft cells to release IL-25. As a group of taste-chemosensory cells, tuft cells are the primary IEC subset expressing taste receptors such as succinate receptor (SUCNR1) and bitter-taste receptors (Tas2rs), which detect the presence of *Tritrichomonas muris* and *T. spiralis* (Schneider et al., 2018; Luo et al., 2019) (**Figure 1**).

In order to allow persistence of the parasite in the host, intestinal parasites, especially *H. polygyrus*, have evolved several mechanisms to negate the function of alarmin cytokines (**Figure 1**). It was noted early that soluble excretory/secretory products of *H. polygyrus* (HES) potently suppress type 2 inflammation (McSorley et al., 2012). Nowadays, two immunomodulatory proteins from HES have been identified by LC-MS/MS, which include *H. polygyrus* alarmin release inhibitor (HpARI) and *H. polygyrus* binds alarmin receptor and inhibits (HpBARI). Both HpARI and HpBARI contain complement control protein (CCP) domains which are present in different phyla including chordates and nematodes. HpARI cannot enter intact cells but can only gain access to the nucleus of necrotic cells, where it binds directly to IL-33 and nuclear DNA via its CCP domains, tethering IL-33 within necrotic cells. HpARI can also directly bind to both mouse and human extracellular IL-33 to prevent IL-33 interact with ST2 on target cells (Osbourn et al., 2017; Chauché et al., 2020). HpBARI binds ST2 and inhibits cell surface detection of ST2, which prevents IL-33-ST2 interactions and blocks IL-33 responses (Vacca et al., 2020).

Alarmin cytokines can also drive immune tolerance of host to the intestinal parasites. The functional response of IL-33 is dictated by its cellular source. Whereas IL-33 derived from IECs elicits host-protective immune responses, IL-33 released from myeloid cells such as DCs suppresses these responses in mice infected with *N. brasiliensis.* DCs use the inducible transmembrane pore-forming protein perforin-2 to deliver IL-33 from the cytoplasm into the extracellular space. DC-derived IL-33 functions to activate the intestinal Treg population through interaction with ST2 (Hung et al., 2020). Moreover, IL-25 induces B1 cell IgE production which blocks *N. brasiliensis* clearance through inhibition of mucosal mast cell activation (Martin et al., 2018).

Alarmin cytokines not only modulate the parasite colonization in intestine, but also drives the adaptive remodeling of intestine after parasite colonization. It is reported that the length of small bowel from mice colonized with *H. polygyrus* or *Tritrichomonas muris* was significantly longer as compared with non-colonized controls. The increased length of intestine was accompanied by a process of crypt fission resulting in duplication of stem cell niches that was prominent in areas in proximity to the colonized parasites. Mechanically, IL-25 dependent amplification of tuft cell-ILC2 circuit is necessary and sufficient to induce adaptive remodeling of the small bowel. Interestingly, succinate, an end product of *Tritrichomonas muris* metabolism, is a potent inducer of this circuit (Schneider et al., 2018).

## Roles of alarmin cytokines in pulmonary parasitic infections

Various nematode larvae need to pass through the lungs before they develop into adults in the guts or other parasitic sites. Such nematodes include *N. brasiliensis*, *S. venezuelensis*, *Litomosoides sigmodontis* (*L. sigmodontis*), and *etc.* Lung is the major site of larvae killing in anamnestic immunity against *N. brasiliensis*. This process needs pulmonary IL-13-producing ILC2 and Th2 cells working in concert to ensure maintenance of M2 macrophages (Bouchery et al., 2015). Th2 cell requirement can be bypassed by administration of IL-2 or IL-33, resulting in expansion of IL-13-producing ILC2 and larval killing (Bouchery et al., 2015). The prior presence of *H. polygyrus* or *T. muris* in the intestines also protects their hosts against migrating *N. brasiliensis* larvae in the lungs by a process involving IL-33-activated Th2 cells that releases IL-5 and recruits activated eosinophils. Importantly, lung immunity remains intact in mice cleared of prior *H. polygyrus* or *T. muris* infection (Filbey et al., 2019). For *L. sigmodontis*, larvae are rapidly cleared from peripheral blood and retained in the lung tissue of mice sensitized with dead larvae in an IL-33-driving eosinophil-dependent manner (Reichwald et al., 2022; Lenz et al., 2024). Besides playing a crucial role in anamnestic immunity, IL-33 is also involved in larvae killing in the primary infection of nematodes. It is reported that IL-33 from type II alveolar epithelial cells aids to killing the infected larvae of *S. venezuelensis* in the lungs by inducing the proliferation of ILC2 and production of IL-5 and IL-13 during the primary infection (Yasuda et al., 2012).

On the other hand, alarmin cytokines are associated with the development of parasite infection-related pulmonary pathology. As described above, IL-33 is a protective factor for hosts to eliminate migrating larvae of *S. venezuelensis* and *L. sigmodontis* in lungs. By contrast, IL-33 also contributes to the development of the airway hyperresponsiveness induced by *S. venezuelensis* and eosinophilic lung disease induced *L. sigmodontis* through the initiation of strong type 2 immune response (Araujo et al., 2016; Lenz et al., 2024). Parasite egg-induced pulmonary granuloma model is a widely used animal model to study the mechanism of schistosome-induced pathology. Using this model, Hams et al. demonstrate a role for IL-25 in the generation of pulmonary granuloma and fibrosis through the induction of IL-13 release from ILC2 (Hams et al., 2014).

## Roles of alarmin cytokines in hepatic parasitic infections

Liver is another common targeted organ for parasite infection, such as *Echinococcus granulosus* (*E. granulosus*), *Echinococcus multilocularis* (*E. multilocularis*), *Clonorchis sinensis* (*C. sinensis*), *Schistosoma mansoni* (*S. mansoni*), *Schistosoma japonicum* (*S. japonicum*) and *Leishmania donovani* (*L*. *donovani*). Emerging evidence suggests that alarmin cytokines may promote the survival or growth of parasites colonized in the livers. It is reported that IL-33 accelerates the growth of larval stage of *E. multilocularis* in the liver putatively through the induction of a tolerogenic microenvironment (Autier et al., 2023). Administration of exogenous IL-33 increases the burdens of *S. japonicum* in infected mice, whereas worm burdens are decreased when blocking IL-33 using neutralizing antibodies (Yu et al., 2015). In experimental models of visceral leishmaniasis, transgenic BALB/c mice with ST2 deficiency exhibit better control of parasite load in the liver. This is associated with an early infiltration of monocytes and neutrophils, as well as a Th1-polarized immune response. Conversely, administering recombinant IL-33 to BALB/c mice leads to a high parasite burden in the liver, accompanied by a suppressed Th1 response and limited infiltration of monocytes and neutrophils (Rostan et al., 2013).

By contrast, alarmin cytokines may contribute to the development of liver injury and fibrosis induced by parasite infections. In the animal models, it is documented that IL-33 contributes to *C. sinensis* induced biliary injuries and fibrosis potentially through orchestration of type 2 immunity (Yan et al., 2022). The role of alarm cytokines in the liver pathology induced by schistosome infection is intensively investigated, however the conclusion is still controversy. ST2 deficiency or blockade of IL-33 using soluble ST2 treatment or neutralizing antibodies resulted in obvious less Th2 cytokine production and Th2-mediated pathology in livers, including marked decreases in granuloma size and fibrosis extent (McHedlidze et al., 2013). The pathogenic effects of IL-33 in hepatic schistosomiasis largely derived from the promotion of the expansion and IL-13 production of ILC2, or induction of the differentiation of M2 macrophages (McHedlidze et al., 2013; Peng et al., 2016). Our group uncovered that hepatic stellate cell is the primary source of IL-33 and that ILC2 is the primary source of IL-13 in the *Schistosoma japonicum* infected mouse livers (He et al., 2018). On the contrary, there were also several evidence which proved that alarmin cytokines had protective effects or had no impacts in schistosomiasis (Bai et al., 2021; Maggi et al., 2021; Mukendi et al., 2021; Maggi et al., 2023). Blocking IL-33 signaling by knockout of *St2* or *Il33* gene is reported to enhance host mortality by modulating granuloma-mediated pathology. The protective effects of IL-33 could stem from inducing thymic involution-associated naive T cell aging, or upregulation of Treg and downregulation of Th17 (Bai et al., 2021; Xu et al., 2022). Furthermore, an independent group showed that individual ablation of TSLP, IL-25, or IL-33/ST2 had no impact on the progression of *Schistosoma mansoni* induced type 2 inflammation or fibrosis, whereas simultaneous disruption of all three mediators resulted in significant reductions in granuloma-associated eosinophils, fibrosis, and IL-13–producing ILC2 (Vannella et al., 2016). These studies seemed to imply that the roles of alarmin cytokines in the initiation of type 2 immunity and pathology induced by schistosome infection were redundant, and single alarmin cytokine might have limited impacts on the disease progression of schistosomiasis.

## Roles of alarmin cytokines in cerebral parasitic infections

Several parasites can effectively infiltrate and infect the human brain, such as *Plasmodium falciparum* (*P. falciparum*), and *Toxoplasma gondii* (*T. gondii*). In the case of cerebral malaria, while the sequestration of parasite-infected red blood cells has been associated with its pathology, it remains uncertain whether this is the direct or sole cause of the clinical syndrome. The development of cerebral malaria likely involves a multifaceted process, encompassing sequestration, inflammation, and endothelial dysfunction within the brain’s microvasculature (Luzolo and Ngoyi, 2019). Owing to their distinct metabolic and immunological characteristics, neurons are frequently susceptible to attack by *T. gondii*. The parasite multiplies inside neurons, leading to neuronal damage through the release of cytokines and chemokines, ultimately resulting in further neurological impairment and disruption of brain metabolism (Elsheikha et al., 2020). In humans and mice, IL-33 is constitutively expressed at high levels within the brain, particularly in stromal cells such as astrocytes and oligodendrocytes (Gadani et al., 2015; Abd Rachman Isnadi et al., 2018). This alarmin is reported to play a crucial role in cerebral malaria and toxoplasmosis. Children with cerebral malaria have elevated plasma and cerebrospinal fluid sST2 levels, which well correlate with serum markers of endothelial activation and neuronal damage and predict neurocognitive impairment (Fernander et al., 2022). In experimental cerebral malaria, infection triggered a dramatic increase of IL-33 expression in oligodendrocytes via ST2 pathway, and ST2-deficient mice were resistant to parasite induced neuropathology associated with attenuated neuroinflammation and exacerbated neurogenesis (Palomo et al., 2015; Reverchon et al., 2017). Surprisingly, administration of exogenous IL-33 prevented the development of cerebral malaria by orchestrating a protective immune response through ILC2, M2 macrophages and Tregs (Besnard et al., 2015). In experimental cerebral toxoplasmosis, IL-33 is expressed by oligodendrocytes and astrocytes during infection and is required for control of parasite burden. Specifically, astrocytes are capable of directly responding to IL-33, thus engaging the peripheral immune system to initiate a Th1 response in controlling the parasites (Jones et al., 2010; Still et al., 2020). Collectively, IL-33 may initiate a protective or detrimental role in cerebral parasite infection dependent on parasite species and IL-33 source. Nevertheless, it is worth pointing out that although IL-25 and TSLP are involved in type 2 immunity and diseases in the brain (Mamuladze and Kipnis, 2023), their functions in cerebral parasitic infections have not been characterized.

## Conclusions

**Figure 2:**
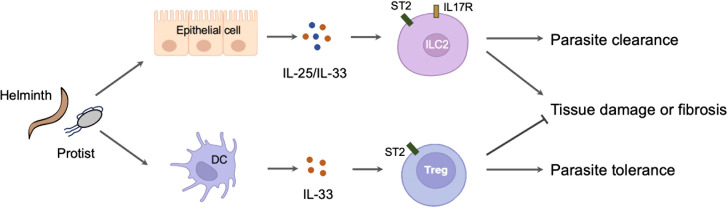
The diverse role of alarmin cytokines in parasitic infections. The invasion of parasites leads to the release of alarmin cytokines from the damaged or stimulated epithelial cells. These cytokines activate ILC2 to mount a protective immunity but also cause host tissue injury or fibrosis. By contrast, the invasion of parasites may simultaneously stimulate the immune cells such as DC to release IL-33. The IL-33 derived from DC activates Treg to mount a tolerance immunity but also prevent host pathology.

The roles of alarmin cytokines in parasitic infections are complex. On the one hand, alarmin cytokines are crucial initiator of Th2 immune response primarily through activating ILC2. This mechanism is important for the expulsion for the adult parasites in intestines and the killing for migrating larvae in lungs. However, strong and long-lasting Th2 immunity induced by alarmin cytokines is also contributes to the development of host tissue injury and fibrosis in lung, liver and brain. In these circumstances, alarmin cytokines are mainly derived from damaged or stimulated epithelial and stromal cells. However, it is still unknown how these cells sense the invasion of parasites and subsequently release the alarmin cytokines. On the other hand, alarmin cytokines especially IL-33 may elicit an immuno-suppressive milieu through inducing the Treg activation or T cell aging. This function promotes host tolerance to parasites but also may inhibit host liver pathology. In this case, IL-33 is released from immune cells such as DCs through an unconventional way (**Figure 2**). Collectively, recent studies have greatly extended our knowledge about the critical and diverse role of alarmin cytokines in parasite infections. These advances may help us to develop novel strategies to prevent or treat parasitic diseases and other diseases. Further studies will be also required to verify the animal model findings in human patients.
